# Apoptotic Cell Death and Inhibition of Wnt/**β**-Catenin Signaling Pathway in Human Colon Cancer Cells by an Active Fraction (HS7) from *Taiwanofungus camphoratus*

**DOI:** 10.1155/2011/750230

**Published:** 2011-03-09

**Authors:** Chi-Tai Yeh, Chih-Jung Yao, Jiann-Long Yan, Shuang-En Chuang, Liang-Ming Lee, Chien-Ming Chen, Chuan-Feng Yeh, Chi-Han Li, Gi-Ming Lai

**Affiliations:** ^1^Cancer Center, Shuang Ho Hospital, Taipei Medical University, Taipei 235, Taiwan; ^2^Center of Excellence for Cancer Research, Taipei Medical University, Taipei 110, Taiwan; ^3^Graduate Institute of Clinical Medicine, Taipei Medical University, Taipei 110, Taiwan; ^4^Cancer Center, Wan Fang Hospital, Taipei Medical University, Taipei 116, Taiwan; ^5^National Institute of Cancer Research, National Health Research Institutes, Miaoli 350, Taiwan

## Abstract

Aberrant activation of Wnt/**β**-catenin signaling plays an important role in the development of colon cancer. HS7 is an active fraction extracted from *Taiwanofungus camphoratus*, which had been widely used as complementary medicine for Taiwan cancer patients in the past decades. In this study, we demonstrated the effects of HS7 on the growth inhibition, apoptosis induction, and Wnt/**β**-catenin signaling suppression in human colon cancer cells. HS7 significantly inhibited proliferation of HT29, HCT116, and SW480 colon cancer cells in a dose- and time-dependent manner. The apoptosis induction was evidenced by DNA fragmentation and subG1 accumulation, which was associated with increased Bax/Bcl-2 ratio, activation of caspase-3 and cleavage of PARP. By using Tcf-dependent luciferase activity assay, HS7 was found to inhibit the **β**-catenin/Tcf transcriptional activities. In addition, HS7 strongly suppressed the binding of Tcf complexes to its DNA-binding site shown in electrophoretic mobility shift assay. This inhibition was further confirmed by the decreased protein levels of Tcf-4 and **β**-catenin. The **β**-catenin/Tcf downstream target genes, such as *survivin*, *c-myc*, *cyclin D1*, *MMP7*, and *MT1-MMP* involved in apoptosis, invasion, and angiogenesis were also diminished as well. These results indicate that *Taiwanofungus camphoratus* may provide a benefit as integrative medicine for the treatment of colon cancer.

## 1. Introduction

As the diet and life styles of the industrialized Western countries are adopted globally, the incidence of colorectal cancer (CRC) is increasing worldwide [1]. It becomes a second most common cancer and the third leading cause of cancer death in Taiwan. Fifty percent of patients diagnosed with CRC will eventually die from the disease, and less than 10% of patients with metastatic CRC survived more than 5 years [2]. Lots of studies have been carried on to search for effective therapeutics targeting on the molecular pathogenesis of CRC. Nowadays, several effective chemotherapeutics and molecular targeting agents, such as bevacizumab and cetuximab, which target VEGFR and EGFR, respectively, had been introduced into current colon cancer therapy. However, most of the advanced patients encountered inevitable relapse. Searching for new therapeutics targeting on other molecular pathway to improve the clinical outcome is urgently needed. 

The Wnt/*β*-catenin signaling pathway plays an important role in both embryogenesis and tumorigenesis. Most sporadic colorectal cancer (CRC) and familial adenomatous polyposis (FAP) have mutated adenomatous polyposis coli (APC) gene. Normal APC protein could downregulate the Wnt signaling pathway through its binding to *β*-catenin and Axin, but most mutated APC proteins in colorectal tumors fail to inhibit Wnt signaling, leading to the overproliferation of tumor cells [3]. Recent researches have implicated that the aberrant Wnt/*β*-catenin signaling pathway plays an important role in several common cancers including liver, lung, and colorectal cancers [4]. The current signaling model of Wnt/*β*-catenin indicates that the binding of Wnt protein to its transmembrane-frizzled receptor could stabilize *β*-catenin by inhibiting the activity of the serine/threonine kinase GSK-3*β* [5]. The GSK-3*β* kinase associated with *β*-catenin in a multiprotein complex, which is composed of the adenomatous polyposis coli tumor suppressor protein APC, axin, conductin, and protein phosphatase 2A. GSK-3*β* phosphorylates the NH2 terminus of *β*-catenin and thereby induces its degradation via the ubiquitin-proteasome pathway [6]. In response to Wnt signaling, GSK-3*β* is inhibited by disheveled proteins, and, therefore, *β*-catenin could escape from phosphorylation. The unphosphorylated *β*-catenin accumulates in the cytoplasm and translocates into the nucleus. In the nucleus, *β*-catenin plays as a transcriptional activator in association with T-cell factor/lymphocyte enhancer factor (Tcf/Lef) family and other transcriptional cofactors [7], by which a variety of target genes including *c-myc*, *cyclin D1*, and *survivin* were subsequently activated [8, 9]. Dysregulation of *β*-catenin signaling is believed to be critical to the early stages of human sporadic colorectal carcinogenesis [10]. The Wnt/*β*-catenin signaling pathway had been proposed as a target for drug discovery [11]. Several substances had been studied for the ability to inhibit this aberrantly activated Wnt/*β*-catenin signaling in colorectal cancer [12, 13]. To find clinically feasible natural substances with this activity but less toxicity is warranted.

*T. camphoratus* (syn. *Antrodia camphorata*) is a *Ganoderma*-like fungus, which belongs to family of Polyporaceae, Basidiomycota, and grows in a unique host, the endemic perennial tree *Cinnamomun kanehirae* (Bull camphor tree) in Taiwan. In the past fifteen years, *T. camphoratus* has been shown to exert antioxidant [14], anti-inflammatory [15], and immunomodulatory activities [16] in a variety of experiments. On the other hand, many studies have demonstrated that *T. camphoratus* exerted multiple biological activities against various cancer cells in the aspects of antiproliferation, apoptosis induction, anti-invasion, and antimetastasis [17–19]. Recently, we have isolated the most potent fraction HS7 from the n-hexane extract of *T. camphoratus *by silica gel chromatography, which appears to possess significant antiproliferative activities against a panel of human cancer cell lines, including lung adenocarcinoma cells (CL1-0), prostate cancer cells (PC3), and hepatocellular carcinoma cells (Hep3B & Huh7). However, the anticancer effects of HS7 on colon cancer cells and the underlying molecular mechanisms remain unclear.

The aim of this study was to address whether the active fraction HS7 isolated from *T. camphoratus* could inhibit the proliferation of human colon cancer cells as well as the Wnt/*β*-catenin signaling pathway. These data may provide important insight for the use of *T. camphoratus* as a potential complementary therapeutic agent for colorectal cancer.

## 2. Methods and Materials

Propidium iodide (PI) and Sulforhodamine B (SRB) were purchased from Sigma-Aldrich Co. (St. Louis, Mo). Trizol reagent, fetal bovine serum (FBS), and trypsin-EDTA (T/E) were obtained from Gibco BRL Life Technologies (Grand Island, NY). The QIAGEN One-tube RT-PCR system and Annexin-V/FITC Apoptosis Kits were obtained from Qiagen Inc. (Valencia, CA) and R&D Systems, Inc. (Minneapolis, MN), respectively. Lipofectamine was purchased from Invitrogen (Rockville, MD). An enhanced chemiluminescence (ECL) kit was purchased from Amersham Corp. (Arlington Heights, IL). Anti-Tcf-4 and anti-active-*β*-catenin antibodies were purchased from Chemicon International, Inc. (Temecula, CA). Anti-Bcl2, anti-Bax, anti-*β*-catenin, and anti-survivin antibodies were purchased from Santa Cruz Biotechnology, Inc. (Santa Cruz, CA), and anticleaved caspase-3 and anti-PARP antibodies were purchased from Cell Signaling Technology, Inc. (Beverly, MA). The plasmids TOPflash and FOPflash were purchased from Upstate Biotechnology, Inc. (Lake Placid, NY). NE-PER nuclear extraction reagent was purchased from Thermo Fisher Scientific Inc. (Rockford, IL). The electrophoretic-mobility shift assay (EMSA) kits were purchased from Panomics, Inc. (Fremont, CA). 

### 2.1. Cell Lines and Cultures

The HT-29, HCT116, and SW480 human colorectal cancer cell lines were obtained from American Type Culture Collection (ATCC). All of the cell lines were maintained in RPMI-1640 culture medium supplemented with 10% fetal bovine serum and 1% Penicillin-Streptomycin-Glutamine (100x) liquid (Gibco). Cells were cultured in an incubator with humidified air at 37°C with 5% CO_2_. The cells were regularly seeded into T-75 flasks with media changes every 3 days. For experiments, cells were grown to 70% confluence, trypsinized with 0.25% trypsin-EDTA, and then replated at a density of 10^5^ per ml in cultured media. As an untreated solvent control, cells were treated with DMSO at a final concentration of <0.05%.

### 2.2. Preparation of Active Fraction HS7 from *T. camphoratus*

The fruiting body-like *T. camphoratus* (voucher number TC-2004-09-001) was cultivated and provided by Well Shine Biotechnology Development Co. (Taipei, Taiwan). Briefly, air-dried ground powder of the cultivated *T. camphoratus* was extracted exhaustively with n-hexane and then separated by silica gel chromatography. Eight fractions (HS1 to HS8) were obtained, and the seventh (HS7) appeared to be the most active fraction on growth inhibition against a screening panel of cancer cell lines.

### 2.3. Sulforhodamine B (SRB) Assay

According to the method described by Vichai and Kirtikara [20], sulforhodamine B (SRB) dye-binding assay was used to determine the antiproliferative effects of HS7 on colon cancer cells. HS7 were dissolved in DMSO before diluting with medium to a final DMSO concentration of <0.05%. The cancer cells were seeded into 96-well plates at density of 3000 cells/well. After 24 hours, the medium was replaced with fresh medium containing different doses of HS7 and then incubated for another 48 hours. The cells were then fixed by TCA (10%) and incubated for 1 hour at 4°C. The plates were then washed 5 times with tap water and air dried. The dried plates were stained with 100 *μ*L of 0.4% (w/v) SRB prepared in 1% (v/v) acetic acid for 10 minutes at room temperature. The plates were washed 4 times with 1% acetic acid to remove unbound dye and then air dried until no moisture was visible. The bound dye was dissolved in 20 mmol/L Tris base (100 *μ*L/well) for 5 minutes on a shaker. Optical densities were measured on a microplate reader (Molecular Devices, Sunnyvale, CA) at 562 nm.

### 2.4. Flow Cytometric Assessment of Apoptosis Using Annexin V Assay

Apoptosis was determined by using a commercially available annexin V apoptosis detection kit and flow cytometry. Following treatment of the colon cancer cells (1 × 10^6^ cells/mL) with different doses of HS7 for 48 hours, cells were harvested and washed twice with 2 mL of ice-cold phosphate-buffered saline (PBS). Cells were then incubated with 100 *μ*L of HEPES buffer containing 2 *μ*L of fluorescein isothiocyanate- (FITC-) conjugated annexin V and 2 *μ*L of propidium iodide (PI) for 15 minutes. After washing the cells of excess reagents, 400 *μ*L of binding buffer was added. The stained cells were immediately analyzed with a FACSCalibur flow cytometer. Approximately 10,000 counts were made for each sample. The percentage distributions of apoptotic cells were calculated by CellQuest software (Becton, Dickinson and Co., San Jose, CA).

### 2.5. Cell Cycle Analysis

Propidium iodide (PI) staining and flow cytometry were used to determine the cell cycle distribution. Cells were treated with different doses of HS7 for 48 hours at 37°C. The treated cells were washed with 5 mL PBS and then harvested using 0.25% Trypsin EDTA and suspended in RPMI at a concentration of 1 × 10^6^ cells/tube. After being washed with PBS and centrifuged at 1200 rpm at 4°C for 5 min, cells were resuspended in 500 *μ*L PBS and fixed with 4.5 mL 70% ethanol followed by gentle vortexing. Cells were allowed to stand overnight at −20°C. Fixed cells were spun down and washed with 5 mL PBS. The cells were then suspended in 500 *μ*L PI (2 *μ*g/mL)/Triton X-100 (0.1% v/v) staining solution with RNase A (200 *μ*g/mL) in the dark and then analyzed by a flow cytometer. Approximately 10,000 counts were made for each sample. The percentage distributions of apoptotic cells were calculated by CellQuest software.

### 2.6. DNA Fragmentation Analysis

The HT29 cells treated with different doses of HS7 were harvested, washed twice with PBS, and then lysed in 100 mL of lysis buffer for 3 hours at 56°C. The cells were treated with RNase A for another hour at 37°C. The DNA was extracted by phenol: chloroform: isoamyl alcohol (v/v/v, 25 : 24 : 1) and analyzed by 1.8% agarose gel electrophoresis. Approximately 2 *μ*g of DNA was loaded into each well. The agarose gels were then run at 50 V for 90 minutes in Tris-borate/EDTA electrophoresis buffer (TBE). The bands were visualized under ultraviolet (UV) light and photographed.

### 2.7. *β*-Catenin/Tcf Transcription Reporter Assay

HT-29 cells were plated in 6-well plates, grown to 80%–90% confluence, and transiently transfected with the plasmids of TOPflash and FOPflash, respectively. TOPflash has 3 copies of the Tcf/Lef binding sites in the upstream of a thymidine kinase (TK) promoter and the firefly luciferase gene. FOPflash has mutated copies of Tcf/Lef sites and is used as control for measuring nonspecific activation of the reporter. All transfections were performed with Lipofectamine and 1.8 *μ*g of TOPflash or FOPflash plasmids. To normalize transfection efficiency, cells were cotransfected with 0.2 *μ*g of the internal control reporter encoding *Renilla reniformis *luciferase driven under the TK promoter. After transfection, cells were incubated in medium with or without HS7 (2.5–25 *μ*g/mL) for 48 hours and then lysed with reporter lysis buffer at harvest. Luciferase activity was determined by using the Dual-Luciferase Assay System kit according to the manufacturer's protocol. The experiments were performed in triplicate, and the results were reported as folds of induction compared with control group after normalization of transfection efficiency.

### 2.8. Immunofluorescence Analysis

For immunofluorescence analysis, cells were plated in 6-well chamber slides for 24 hours before treatment with 10 *μ*g/mL of HS7 for 48 hours. Afterwards, cells were fixed in 2% paraformaldehyde for 10 minutes at room temperature, permeabilized with 0.1% Triton X-100 in 0.01 M PBS pH 7.4 containing 0.2% bovine serum albumin, air dried, and rehydrated in PBS. Cells were then incubated with a rabbit polyclonal antibody against *β*-catenin (1 : 500) diluted in PBS containing 3% bovine serum albumin for 2 hours at room temperature. Negative controls were performed by omitting the primary antibody. After washing with PBS twice for 10 minutes, an antirabbit IgG PE-conjugated secondary antibody (1 : 500) diluted in PBS was added and incubated for 1 hour at room temperature. Cells were then washed by PBS and then mounted using Vectashield mounting medium with 4′,6-diamidino-2-phenylindole (DAPI) to counterstain DNA. Cells were observed using a Zeiss Axiophot fluorescence microscope. Microphotographs were acquired using an AxioCam MRc digital video camera and an Axiovision Zeiss software (Carl Zeiss, Inc., Jena, Germany).

### 2.9. Preparation of Nuclear Extracts

To obtain nuclear extracts lysates, the HS7-treated and -untreated cells were washed twice with ice-cold 1 × PBS and lysed using NE-PER nuclear extraction reagents. The protein concentrations of all the extracts were determined by the bicinchoninic acid protein assay (Pierce, Rockford, IL) with bovine serum albumin as the standard. Nuclear extracts were stored at −70°C until used.

### 2.10. Electrophoretic Mobility Shift Assay (EMSA)

Two to five *μ*g of nuclear extract was used for EMSA according to the manufacturer's instructions. Nuclear extracts containing equal amounts of protein for each sample were incubated with poly (dI-dC) (1 *μ*g/*μ*L) for 5 minutes, followed by the addition of binding buffer and biotinylated oligo (10 ng/*μ*L). After electrophoresis, gels were transferred to nylon membranes. Transferred oligos were immobilized by UV crosslinking for 3 minutes. For detection of bound oligos, membranes were blocked using blocking buffer followed by the addition of Streptavidin-HRP, and blots were developed by ECL according to the manufacturer's instructions. Commercially available biotinylated oligonucleotide encoding the Tcf/Lef motif 5′-CCTTTGATCTTCCTTTGATCTT-3′ was used as a canonical probe (Panomics, AY-11149P).

### 2.11. RNA Extraction and RT-PCR

The expression levels of *survivin*, *c-myc*, *cyclinD1*, *MMP7*, and *MT1-MMP* were quantified by semiquantitative reverse transcription-polymerase chain reaction (RT-PCR) analysis, using *GAPDH* mRNA as an internal standard. HT29 cells (1 × 10^6^ in 10 mL medium) were plated in 100 mm tissue culture dishes. After plating for 24 hours, HT29 cells were treated with different doses of HS7 for 48 h. At harvest, cellular RNA was extracted with a TRIzol RNA isolation kit as described in the manufacturer's manual. RNA concentration and purity were determined based on measurement of the absorbance at 260 nm and 280 nm. After adding RNase inhibitor (20 U), the total RNA was stored at −70°C. The sense and antisense primer sequences used were as follows: 

*survivin*: 5′-ATGGGTGCCCCGACGTTGC-3′, 5′-TCAATCCATGGCAGCCAGCTG-3′;

*c-Myc*: 5′-CGAGCTGCTGGGAGGAGACAT-3′, 5′-AGCCGCCCACTTTTGACAGG-3′;

*cyclinD1*: 5′-CTACAGGGGAGTTTTGTTGA-3′, 5′-GGTAGTAGGACAGGAAGTTG-3′;

*MMP7*: 5′-CCTACAGGATCGTATCATAT-3′, 5′- GGAACAGTGCTTATCAATTC-3′;

*MT1-MMP*: 5′-TCGGCCCAAAGCAGCAGCTTC-3′, 5′-CTTCATGGTGTCTGCATCAGC-3′;

*GADPH*: 5′-CAAAAGGGTCATCATCTCTGC-3′, 5′-GAGGGGCCACACAGTCTTC-3′,

respectively. From each sample, 250 ng of RNA was reverse-transcribed, using 200 U of SuperScript II RNase-H reverse transcriptase, 20 U of RNase inhibitor, 0.6 mM of dNTP, and 0.5 mg/mL of oligo (dT). PCR analyses then were performed on the aliquots of the cDNA preparations to detect *survivin*, *c-Myc*, *cyclinD1*, *MMP7*, *MT1-MMP*, and *GAPDH* (as an internal standard) genes expression, using the FailSafe PCR system (Epicenter Technologies, Madison, WI). The reactions took place in a volume of 50 *μ*L, containing a final concentration of 50 mmol/L Tris-HCl, (pH 8.3), 50 mmol/L KCl, 1.5 mmol/L MnCl2, 0.2 mmol/L dNTP, 2 U of Taq DNA polymerase, and 50 pmol of primers. After initial denaturation for 2 minutes at 95°C, 26 cycles of amplification (at 95°C for 1 min, 60°C for 1 min, and 72°C for 1.5 min) were performed, followed by a 7-minute extension at 72°C.

### 2.12. Analysis of PCR Products

A 10 *μ*L aliquot from each PCR reaction was electrophoresed in a 1.8% agarose gel containing 0.2 mg/mL ethidium bromide. The expression level of* survivin* was normalized by the corresponding *GAPDH *signal from the same gel and expressed as the ratio of *survivin/GAPDH*.

### 2.13. Western Blotting

At harvest, the HS7-treated and -untreated cells were washed twice with PBS (pH 7.0), and the total proteins were extracted by adding 200 *μ*L of cold lysis buffer to the cell pellets on ice for 30 minutes. This was followed by centrifugation at 10,000× g for 30 minutes at 4°C. Western blotting was performed according to the method of Yeh and Yen [21]. The membrane was further incubated overnight at 4°C with respective specific antibodies against caspase-3 (1 : 1000), PARP (1 : 1000), Bcl-2 (1 : 1000), Bax (1 : 1000), active *β*-catenin (1 : 1000), Tcf-4 (1 : 1000), survivin (1 : 1000), and *β*-actin (1 : 5000). After incubation with primary antibodies, the membrane was washed with TBST 3 times. The membrane was then incubated with horseradish peroxidase-labeled secondary antibody for 45 minutes at room temperature and washed with TBST 3 times. Final detection was performed with enhanced chemiluminescence (ECL) Western blotting reagents.

### 2.14. Statistical Analysis

Each experiment was performed in triplicate and repeated 3 times. The results were expressed as means ± SD. The significance of differences between HS7-treated and vehicle-treated control groups was analyzed by *t*-test as appropriate. “∗” indicates that the values are significantly different from the control (*P < .05; **P < .01).

## 3. Results

### 3.1. Growth Inhibition and Apoptosis Induction of Human Colon Cancer Cells by HS7 Fraction from *T. camphoratus*

**Figure 1:**
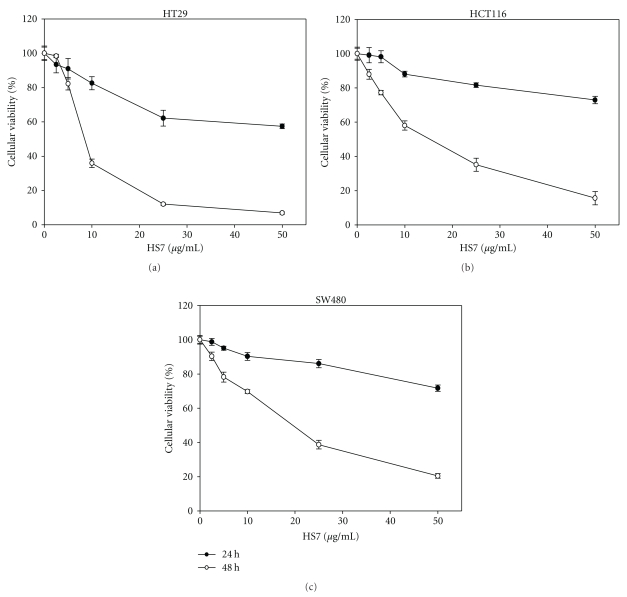
HS7 inhibited colon cancer cell proliferation in a dose- and time-dependent manner in 3 cell lines: (a) HT29, (b) HCT116, and (c) SW480. Cells were treated with HS7 at a final concentration of 0–50 *μ*g/mL for 24 h and 48 h (the control group was treated with DMSO at concentration of <0.05%). Cell proliferation was determined by SRB assay. Results for the means of cell proliferation (%) ± SD of triplicate measurements are shown (*P < .01 compared with control).

**Figure 2:**
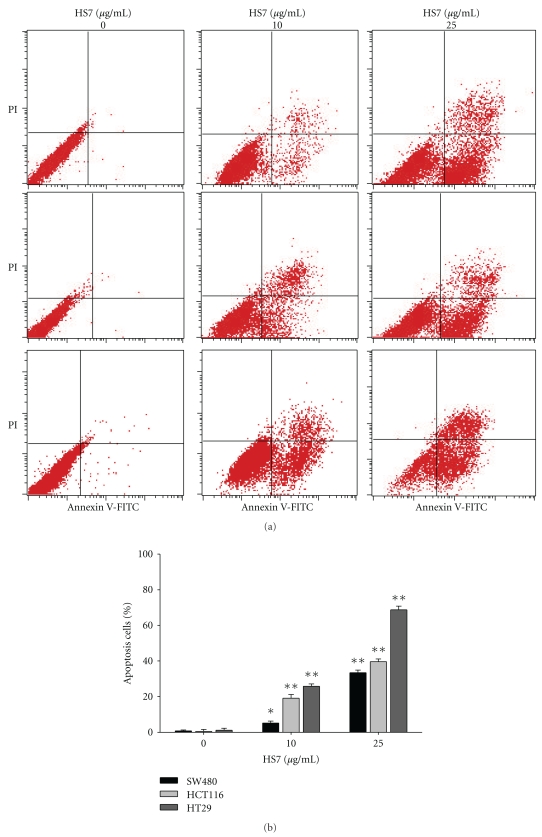
HS7 induced apoptosis in human colon cancer cells. (a) SW480, HCT116, and HT29 cells were treated with a range of HS7 (0, 10, and 25 *μ*g/mL) for 48 h. Cells then were stained with annexin V/propidium iodide (PI) and analyzed by flow cytometry. Apoptotic cells were localized in the lower right (early apoptosis) and upper right (late apoptosis) quadrants of the dot-plot graph using annexin V versus PI. (b) Bar graphs represent the mean values of triplicate measurements ± SD. *P < .05; **P < .01, compared with control.

**Figure 3:**
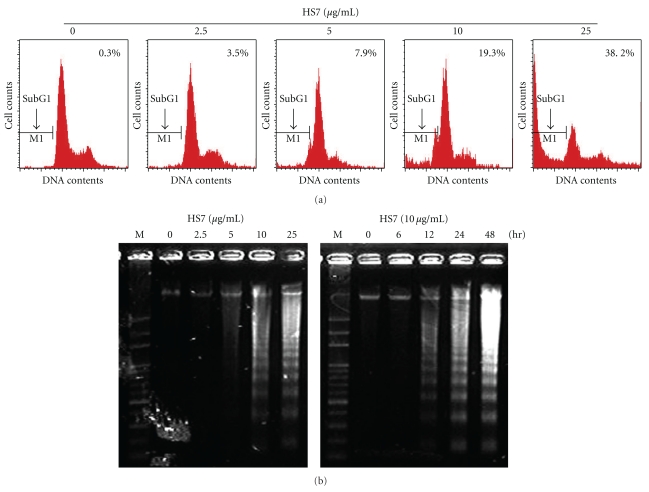
Apoptosis induced by HS7 in HT29 cells. (a) HS7 increased the subG1 population in HT29 cells. HT29 cells were grown in the absence (control) or presence of HS7 (2.5–25 *μ*g/mL) for 48 h, stained with propidium iodide (PI), and analyzed by flow cytometry for DNA content. Arrows indicate predicted location of fragmented DNA or subG1 population. (b) DNA fragmentation of HT29 cells exposed to HS7 is shown. Genomic DNA was extracted from HS7-treated HT29 cells and separated on 1.8% agarose gels.

The growth inhibitions of human colon cancer cells by HS7 were shown in Figure 1. HS7 had significant antiproliferative effects on HT-29, HCT-116, and SW-480 cells in a dose- and time-dependent manner. After 48 h incubation, the growth inhibition was most noticeable in HT-29 cells with IC50 of 9.5 *μ*g/mL. In contrast, the IC50 for HT-116 and SW-480 cells were 15.1 and 19.4 *μ*g/mL, respectively. As shown in Figure 2, HS7 increased the apoptosis in a dose-dependent manner in HT29, HCT116, and SW480 cells, including both the early apoptosis (Annexin V-FITC+/PI−) and the late apoptosis (Annexin V-FITC+/PI+). At concentration of 25 *μ*g/mL for 48 h, the amount of annexin V-FITC-positive cells were 68.7 ± 1.1%, 39.6 ± 1.2%, and 33.5 ± 1.7% in HT29, HCT116, and SW480 cells, respectively, compared to control (less than 0.2%). In addition, it was noted that there was a remarkable dose-dependent accumulation of subG1 peak in HS7-treated HT29 cells when compared with the untreated group (Figure 3(a)). Furthermore, agarose-gel electrophoresis of HS7-treated chromosomal DNA showed a ladder-like pattern of DNA fragments in a time- and dose-dependent manner (Figure 3(b)). Our results suggested that the apoptosis induction by HS7 might play a substantial role on the growth inhibition of HT29 cells. 

**Figure 4:**
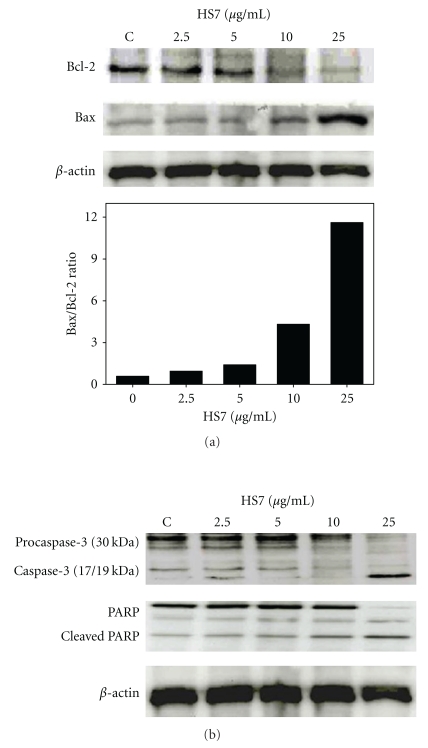
HS7 regulated expression of specific proteins in HT29 cells involved in apoptosis. (a) Protein expression of Bax and Bcl-2 and Bax/Bcl-2 ratio were shown. HT-29 cells were exposed to 2.5–25 *μ*g/mL of HS7 for 48 h. Bax and Bcl-2 expression was determined by Western blot. Relative changes in Bcl-2 and Bax protein bands were measured using densitometric analysis. (b) HS7 induced caspase-3 activation and cleaved PARP in HT29 cells. Typical results from 3 independent experiments were shown.

Since HS7 induced significant apoptosis in HT29 cells, we subsequently studied the cellular proteins involved in apoptosis induction. The Bcl-2 and Bax, two important members of the Bcl-2 family involved in the mitochondrial apoptosis pathway, and the downstream elements caspase-3 and PARP were examined. In HT29 cells, as shown in Figure 4(a), HS7 caused a significant upregulation of Bax and downregulation of Bcl-2 protein levels, leading to dose-dependent increase of the Bax/Bcl-2 ratio. Furthermore, the dose-dependent increased fragments of 17/19 kDa caspase-3 and 85 kDa-cleaved PARP further confirmed the apoptosis induction (Figure 4(b)). These results suggest that the mitochondria-dependent pathway may participate in the HS7-induced apoptosis of HT29 cells.

### 3.2. HS7 Inhibits *β*-Catenin/Tcf Pathway in Human Colon Cancer Cells

**Figure 5:**
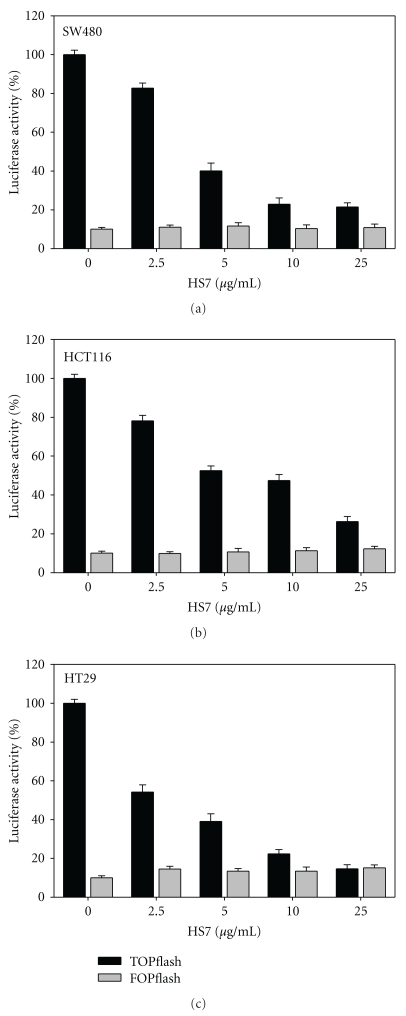
HS7 inhibited the transcriptional activity of beta-catenin/Tcf in colon cell lines. (a) SW480, (b) HCT116, and (c) HT29 cells were transiently cotransfected with TOPflash or FOPflash and Renilla pRL-TK reporter plasmids by Lipofectamine 2000. Six hours after transfection, cells were treated with 0, 2.5, 5, 10, and 25 *μ*g/mL HS7 for 48 h. Relative luciferase activity was normalized by transfection efficiency as determined by Renilla luciferase activity.

 To elucidate the role of HS7 on the dysregulated Wnt/*β*-catenin of colorectal cancer cells, we examined the activity of *β*-catenin/Tcf pathway in HS7-treated cells. The HT29, HCT116, and SW480 cells, having a constitutively active transcriptional activity of *β*-catenin/Tcf, were transiently transfected with the reporter plasmid TOPflash or FOPflash. The transfection efficiency was normalized by the cotransfected Renilla expression vector. As shown in Figure 5, HS7 dose-dependently reduced the Tcf-dependent luciferase activity (TOPflash) of all 3 colon cancer cells after 48 h of treatment, while the FOPflash activity, a mutant of *β*-catenin/Tcf binding, remained unchanged. In HT29 cells, the Tcf transcriptional activities were reduced to 54 ± 3%, 39 ± 4%, 22 ± 2%, and 14 ± 2% of the control by HS7 at dose of 2.5 *μ*g/mL, 5 *μ*g/mL, 10 *μ*g/mL, and 25 *μ*g/mL, respectively. This indicates that the transcriptional activity of *β*-catenin/Tcf could be inhibited by HS7 and thus suppresses the Wnt/*β*-catenin signaling pathway.

**Figure 6:**
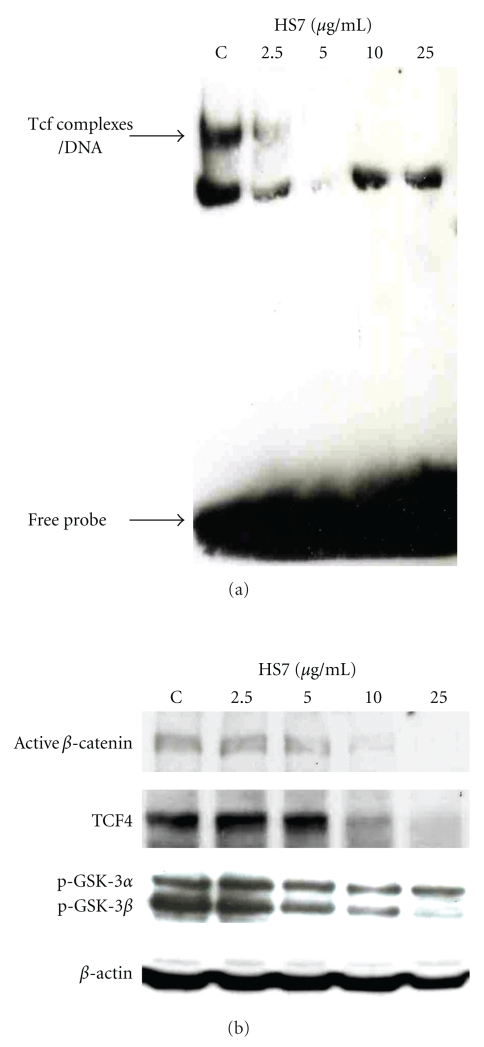
HS7 dose-dependently decreased the binding of Tcf complex to DNA and downregulated the protein levels of active *β*-catenin, Tcf-4, and p-GSK3*α*/*β*. (a) HT-29 cells were treated with HS7 at the indicated concentrations for 48 h, and nuclear extracts were isolated. The electrophoretic-mobility gel shift assay (EMSA) was performed with 5 *μ*g nuclear extracts. (b) HT-29 cells were treated with HS7 (0–25 *μ*g/mL) for 48 h, the changes of active *β*-catenin, Tcf-4, and p-GSK3*α*/*β* protein levels were then analyzed by Western blot. *β*-actin was used as internal control. All pictures are representative of two independent experiments.

Binding to its DNA response element is the most important step for the biological function of transcription factor. Therefore, we performed electrophoretic mobility shift assay (EMSA) to investigate whether the inhibitory effect of HS7 on the transcriptional activity of *β*-catenin/Tcf complex was caused by the disruption of its DNA binding. In HT29 cells, Tcf complexes had substantial binding activity to its DNA response elements. After 48 h treatment, such binding was decreased by HS7 in a dose-dependent manner (Figure 6(a)). At the same dose range, the active *β*-catenin, Tcf-4, and inactived/phosphorylated GSK3*α*/*β* protein levels were markedly decreased (Figure 6(b)), which would result in the reduced binding of *β*-catenin/Tcf complex to its DNA response elements.

**Figure 7:**
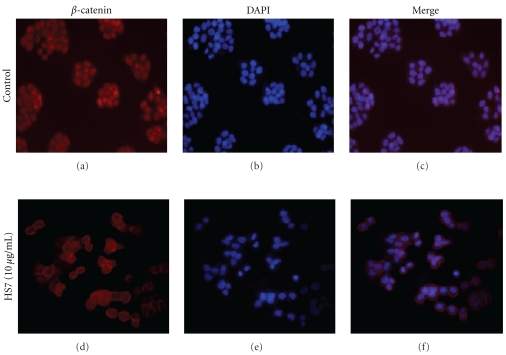
Effect of HS7 on cellular localization of *β*-catenin in HT-29 cells was shown. Cellular localization of *β*-catenin was evaluated by indirect immunofluorescence using a monoclonal antibody that labeled *β*-catenin. Immunofluorescence labeling of *β*-catenin in cells treated with vehicle (a) or 10 *μ*g/mL HS7 (c) for 48 h were shown (red). Nuclei in cells treated with vehicle (b) or 10 *μ*g/mL HS7 (d) for 48 h were counterstained with 4′, 6-diamidino-2-phenylindole (blue). Merged images show that the *β*-catenin protein located at cell-cell contacts in HS7-treated cells was significantly increased as compared to control (e and f). Magnification for each representative picture: 200x (from 3 separate experiments).

 To determine whether the HS7 suppressed *β*-catenin-mediated transcription accompanied with decreasing the nuclear *β*-catenin translocation, we investigated the *β*-catenin subcellular localization in HS7-treated HT29 cells by indirect immunofluorescence staining. As shown in Figure 7(a), the *β*-catenin was present in all cellular localizations and preferentially accumulated in the nucleus in the control cells, which was confirmed by DAPI staining (Figure 7(b)). In contrast, after treatment with HS7 at dose of 10 *μ*g/mL for 48 h, the localization of *β*-catenin decreased at nuclei but increased at cell-cell contacts (Figures 7(c) and 7(d)).

### 3.3. HS7 Suppresses the Expression of *β*-Catenin/Tcf Pathway Downstream Target Genes

**Figure 8:**
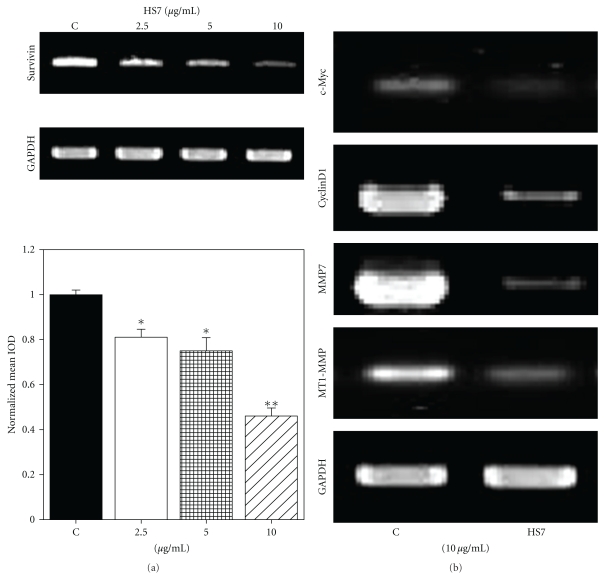
Inhibitory effects of HS7 on the expressions of Tcf/*β*-catenin downstream genes in HT29 cells. (a) Cells were treated with HS7 (2.5–10 *μ*g/mL) for 48 h. Survivin mRNA expression was evaluated by RT-PCR. Densitometric analysis of RT-PCR results normalized to GAPDH was shown as bar graph. Results represent the mean of 3 experiments ± SD. (b) Cells were exposed to 10 *μ*g/mL HS7 for 48 h, and the expressions of c-Myc, CyclinD1, MMP7, and MT1-MMP mRNAs were evaluated by RT-PCR. *P < .05; **P < .01 compared with control.

Since *survivin*, *c-Myc*, *cyclin D1*, matrix metalloproteinase-7 (*MMP7*), and membrane type I MMP (*MT1-MMP*) are the downstream target genes of *β*-catenin/Tcf pathway, we examined whether HS7 could downregulate the expressions of those genes in HT29 cells. At the dose of 2.5–10 *μ*g/mL, HS7 was able to inhibit *survivin* mRNA expression in a dose-dependent manner (Figure 8(a)). Similarly, after 48 h of treatment with 10 *μ*g/mL HS7, the expressions of *c-Myc*, *cyclin D1*, *MMP7*, and *MT1-MMP* were also significantly downregulated (Figure 8(b)). 

**Figure 9:**
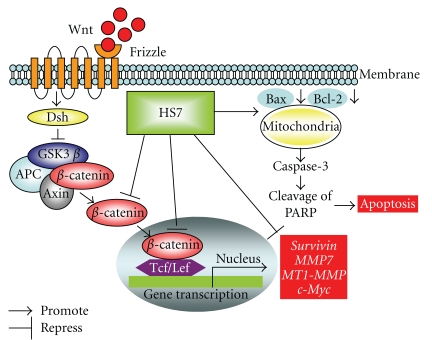
Hypothetical diaphragm of HS7 induced apoptosis and inhibition of Wnt/*β*-catenin signaling pathway in HT-29 colon cancer cells. The arrow indicates promotion, and the T-shaped bar indicates repression. Dsh: dishevelled protein, GSK-3*β*: glycogen synthase kinase-3 beta, APC: adenomatous polyposis coli protein, Tcf/Lef: T-cell factor/lymphocyte enhancer factor family, PARP: poly (ADP-ribose) polymerase.

The hypothetical diaphragm describing the molecular mechanisms responsible for anticancer effects of HS7 on colon cancer cell was illustrated in Figure 9.

## 4. Discussion

 The induction of apoptosis by crude extract of *T. camphoratus* had been reported in several types of cancer cells such as prostate cancer and hepatoma cells [22, 23]. Its effects on the apoptosis modulating proteins, such as Bcl-2, Bax, and Bid, had been demonstrated in Hep 3B hepatoma cells [23]. In accordance with those studies, our data also showed the increased Bax/Bcl-2 ratio, activated Caspase-3, and cleaved PARP in HS7-treated HT-29 colon cancer cells. The *T. camphoratus*-induced apoptosis was significant in wild-type p53 expressing prostate cancer cells LNCaP but limited in null p53 prostate cancer cells PC3 [22]. However, in our data, HS7 induced significant apoptosis not only in wild-type p53 expressing HCT116 cells but also in p53 mutant HT29 and SW480 cells. The correlation of p53 defective status with apoptosis induced by *T. camphoratus* may need further investigation.

Many natural triterpenes had been reported able to induce apoptosis in cancer cells. Based on our previous study in SW480 and HT-29 colon cancer cells, several triterpenes isolated from *T. camphoratus* also exerted the apoptosis inducing effects [24]. To date, two known phytochemicals are present in the active fraction HS7 extracted from *T. camphorates*. One is 15-*α*-acetyl-dehydrosulphurenic acid [25] and another is antroquinonol [26]. In addition, we had isolated a novel compound named P7b from HS7, which is more potent than antroquinonol in anticancer activities. The identification of the chemical structure of P7b is ongoing now. 

In this study, we also demonstrated the inhibitory effects of HS7 on the Wnt/*β*-catenin signaling pathway of HT-29 colon cancer cells. Based on the EMSA and luciferase reporter assays, both the DNA binding and the transcription activity of *β*-catenin/Tcf complex were effectively inhibited by HS7. These effects were further confirmed by the suppressed expression of *β*-catenin/Tcf downstream target genes, such as *survivin*, *c-Myc*, *cyclin D1*,* MMP7*, and *MT1-MMP* in HS7-treated HT-29 cells. This inhibition of Wnt/*β*-catenin signaling pathway may result from the decreased nuclear translocation of *β*-catenin and the downregulated protein levels of both Tcf-4 and active *β*-catenin. This is the first study demonstrating the inhibitory effects of *T. camphoratus* on the Wnt/*β*-catenin signaling pathway of colon cancer cells. 

The level of *β*-catenin in cells is tightly controlled by its degradation complex composed of Axin, APC, GSK-3*β*, and *β*-catenin, in which GSK-3*β* phosphorylates *β*-catenin and thus triggers its ubiquitination and subsequent proteasomal degradation. As *β*-catenin negative regulator GSK3*β* is inactivated by phosphorylation, the HS7-induced decrease of p-GSK3*β* might contribute to its effects on Wnt/*β*-catenin pathway inhibition. Taken together, the above findings provide evidence for GSK-3*β* dephosphorylation in HS7-induced degradation of *β*-catenin in colon cancer cells. 

Several genes activated by dysregulated *β*-catenin, such as *c-myc* and *survivin*, are regarded as apoptosis regulators. The *β*-catenin has become further implicated in the regulation of apoptosis recently. Steinhusen et al. reported the apoptosis-induced cleavage of *β*-catenin by caspase-3; however, they proposed that the proteolytic cleavage of *β*-catenin might not be simply an effect of apoptosis [27]. More recently, the caspase-mediated cleavage of *β*-catenin was demonstrated to precede drug-induced apoptosis in resistant cancer cells, indicating the potential of *β*-catenin as a promising new target of drug-induced apoptosis [28]. In our results, HS7 markedly inhibited the *β*-catenin/Tcf-dependent luciferase activity at dose of 5 *μ*g/mL, whereas the caspase-3 was markedly cleaved and activated at 25 *μ*g/mL. The inhibition of Wnt/*β*-catenin signaling pathway might be an important part of HS7-induced apoptosis pathway rather than just a downstream effect of apoptosis. Similarly, our results showed that *β*-catenin protein mainly located at cell-cell contacts after treatment with HS7 (10 *μ*g/mL) in colon cancer cells. The nuclear staining of *β*-catenin was markedly reduced as compared with that in control group. Therefore, this inhibition of *β*-catenin-induced transcription might result in the apoptosis of colon cancer cells. 

Aspirin and other nonsteroidal anti-inflammatory drugs (NSAIDs), being used as chemopreventive agents against CRC, had been found to inhibit the Wnt/*β*-catenin signaling pathway [11]. Both aspirin and indomethacin could decrease the transcriptional activity of *β*-catenin/Tcf-responsive genes [29]. Sulindac is able to suppress the nuclear *β*-catenin localization and *β*-catenin/Tcf-regulated genes [30]. Celecoxib, the only one NSAID approved by Food and Drug Administration in the USA for the treatment of familial adenomatous polyposis (FAP), was also found to inhibit the Wnt/*β*-catenin signaling pathway in colon cancer cells [31]. However, most NSAIDs except celecoxib may cause adverse effects such as bleeding and the gastric mucosa damage due to their more effects on COX-1 inhibition. Nevertheless, the celecoxib had side effects on the risk of cardiovascular diseases [32]. Thus, searching for alternative agents other than NSAIDs is needed for the prevention and treatment of CRC.

Regarding the profound suppressing effects on the Wnt/*β*-catenin pathway of colon cancer cells, HS7 may serve as an alternative CRC preventing dietary supplement in addition to NSAIDs. Similar to HS7, other natural dietary substances such as epigallocatechin-3-gallate (EGCG), curcumin, and resveratrol had also been demonstrated to exert activities on Wnt/*β*-catenin pathway inhibition [33–35]. Combined use of these active natural substances may have synergistic effects and become a good strategy to achieve better clinical effectiveness. Therefore, further preclinical studies on such combinations are warranted. 

Wnt/*β*-catenin signaling also appears to regulate cancer cell invasion, metastasis, and angiogenesis [36]. Holcombe et al. had demonstrated that the expression of Wnt family ligands correlated with the invasion of colon cancer [37]. Recently, Brabletz et al. reported that *β*-catenin/Tcf-regulated genes, such as *MMP7* and *MT1-MMP,* were involved in the regulation of cell growth, invasion, and angiogenesis of colorectal cancer [38, 39]. In particular, *MMP-7* has been shown to be overexpressed in 75% of human colorectal cancers and correlate with stages of disease and/or prognosis [40]. Similarly, *MT1-MMP* is overexpressed in different tumors, including colon cancer and correlates with the invasiveness and metastasis [41]. Furthermore, Peng et al. demonstrated that *T. camphoratus* suppressed the active MMP-9 levels correlated with the inhibition of transwell motility of T24 bladder cancer cells [42]. Regarding the substantial inhibitory effects of HS7 on Wnt/*β*-catenin signaling and its downstream *MMP7* and *MT1-MMP *mRNAs expression, it indicates the potential use of HS7 as anti-invasion and antiangiogenesis agent against colon cancer cells. 

The importance of Wnt/*β*-catenin signaling is also highlighted in modulating stemness, proliferation, and differentiation in several adult stem cell niches, such as hair follicles, mammary gland, intestinal crypt, and hematopoietic tissues [43]. In addition, the Wnt/*β*-catenin signaling also plays an important role in cancer stem cells [44]. Expression of stabilized *β*-catenin promotes the self-renewal of hepatic stem/progenitor cells and leads to tumorigenesis in the liver [45]. Recently, we have successfully isolated cancer stem-like side population (SP) cells from Huh 7 hepatoma cell line and found that HS7 could reduce the proportion of these SP cells accompanied with downregulation of Wnt/*β*-catenin signaling and thereby suppressing the tumorigenicity of SP cells on NOD/SCID mice (data not shown), indicating that HS7 may have effects on the elimination of cancer stem cells.

In addition to the remarkable effects of HS7 on apoptosis induction and inhibition of Wnt/*β*-catenin signaling pathway, further investigations to explore its effects on other signaling pathways as well as the aspects of antiangiogenesis, anti-invasion, and elimination of cancer stem cells are strongly recommended. The effects of HS7 shown in this study may provide important insight for the use of *T. camphoratus* as a potential complementary and integrated medicine for the treatment of colorectal cancer.
